# The Juridical Society, and the Criminal Responsibility of the Insane

**Published:** 1858-01-01

**Authors:** 


### Art. X.—THE JURIDICAL SOCIETY, AND THE CRIMINAL RESPONSIBILITY OF THE INSANE.

(From a Correspondent.)

No well-wisher to his species can fail to rejoice that the ice which divides the medical and legal professions on the subject of the Criminal Responsibility of the Insane, has at length been broken. It were hopeless to expect that the imperfect expositions of what they imagine to be the views entertained by medical men on the subject of insanity, that barristers are in the habit of making on occasions when they defend prisoners charged with criminal offences, could ever dissipate the dense obscurity which surrounds these subjects in the public mind. The legal profession generally, and especially the judges, have so little practical acquaintance with insanity, that their minds are absolutely unable to comprehend vast truths which are familiar enough to medical men. Examinations in courts of justice are peculiarly unfavourable to the diffusion of just ideas on these matters, and the medical witness consequently gives his testimony amidst an amount of prejudice, arising from ignorance, which is too often fatal to the best interests of humanity and of justice. The existence, then, of the Juridical Society, composed as it is of the most eminent members of the legal profession, and of a few other persons distinguished for their attainments in literature and jurisprudence, is a fact calling for devout thankfulness on the part of those who look for free discussion as one of the best means of eliciting truth and of diffusing knowledge. Monday, the 14th of December, will henceforth be considered a great day in the history of criminal jurisprudence; for upon it there met together a large number of eminent lawyers, and eminent medical men, to discuss the subject of the criminal responsibility of lunatics. Dr. Forbes Winslow read a paper on "the Doctrine of Responsibility in Cases of Insanity connected with alleged criminal acts," which was followed by an interesting discussion, in which the Vice-Chancellor, Sir John Stuart, who was in the chair, and Mr. Baron Bramwell, took an active part. We desire, in the following remarks, to direct the attention of our readers to what we conceive to be grave and fundamental errors in the reasoning, especially of Mr. Baron Bramwell, on that occasion—because, if our views are correct, the doctrines laid down by the learned Judge strike at the root of the theory upon which all punishment is based. If Baron Bramwell be wrong, then are his errors the most serious and vital that it is possible to conceive in one whose function it is indeed to administer the law, but who would very imperfectly fulfil the duties of an English Judge if he did not also endeavour to improve the law. We have no reason to suppose that the learned Judge is not sincerely desirous to amend what may be defective in the laws; on the contrary, his frequent attendance at the meetings of the Juridical Society shows a desire to advance the science of jurisprudence, which cannot be too highly appreciated by the public. We shall endeavour to reproduce the chief arguments of Baron Bramwell with scrupulous accuracy, and we have no fear of not doing so correctly, for his words fell with painful distinctness upon our ear, and have been very faithfully reported in some of the daily papers. It would be almost an insult to assure the learned Judge that our remarks are made in a spirit of sincere and profound respect for his judicial position and attainments. The truth is that at which we aim, and we are sure it is the truth alone that Baron Bramwell seeks.

The learned Judge observed that "the question to be discussed was not the relative amount of pity which we should feel for the sane or the insane, but how is the law to deal with the commission of an act which it prohibits? To solve this question, it is necessary to go back to the true theory of punishment, which is, that pain being in itself an evil, society has no right to inflict it upon an individual, except for the purpose of preventing crime, by the fear of it on the individual punished, and by the spectacle of it on the rest of the community. The *certainty*, therefore, with which punishment follows crime is of the last importance in teaching men to respect the law, and to abstain from breaking it; for since the law threatens all mankind, it would be a mere *brutum fulmen* if it did not also punish those who violate it. The madman, amongst others, is threatened by the law; why then should he escape if he infringes the law? and why destroy that certainty of punishment following crime which is the very essence of its preventive power? For his part, he could conceive an argument being maintained to show that even idiots should be punished when they break the law; but in such an opinion, if held by any one, he did not share. If you do not punish the madman, you hold out a premium to the commission of crime; for every man would calculate that he would be fortunate enough to escape by some one proving that he was mad, on the same principle as that on which people lead a forlorn hope, or put into a lottery, not calculating the chances against them, but trusting that they will be the fortunate ones to survive or to win the prize." Baron Bramwell made some further remarks in reference to cases which he had tried within the last two years, and also enunciated the astounding opinion that he doubted the existence of moral qualities in the mind. Our concern, however, just now is with the theory of punishment set forth in the above quotation from his speech. It is not the first time that we have heard these doctrines; but we believe them to be utterly erroneous and untenable, and to arise chiefly in consequence of persons confounding together punishments following infractions of the physical laws of nature with those which follow violations of the laws of society. The two classes of penalties stand upon totally different grounds, although it is not uncommon to hear the remark that crime would be most effectually put down if punishment followed on its commission with as much *certainty* as it does on the breakage of physical laws. It is not the mere certainty of a particular punishment following a particular crime that constitutes the efficacy of the penal code; but it is the certainty that *a right measure of punishment, adapted to the peculiarities of each individual case,* will follow upon conviction. Public opinion must go along with and support the administration of justice, or that very element of uncertainty which Baron Bramwell so highly deprecates is at once, and as a direct consequence, introduced into the working of the law. Severe and unjustifiable penalties carry with them the elements of their own failure; they are conceived in ignorance, and cannot stand the light of day. It is for this reason that the numerous executions which formerly disgraced the history of jurisprudence in this country were found wholly inefficacious in diminishing crime. Juries would not convict persons who were proved to have forged or uttered one-pound notes, or stolen to a trifling amount from a dwelling-house; and although the Judges recorded the sentences of death, and the Government carried them out with unsparing severity whenever they had the opportunity, the very *certainty* with which an unjust penalty would be inflicted on conviction, proved the safeguard to prisoners, and the means of their escaping from any punishment at all. Whence, then, arises a result as uniform in the history of the laws as we must suppose it is surprising to Baron Bramwell? Why, from what else but from that very *moral sense* inherent in all mankind, the existence of which the learned Judge calls in question? It is because society feels and knows that all its punishments are inflicted under direct and fearful responsibility, and must be proportioned to the degree of guilt of each criminal, lest they become, not punishments, but acts of cruelty, and crimes themselves. In this respect they differ altogether from the blind unreasoning penalties that attach to violations of physical laws. If a sane man or an idiot, a rèasoning being or a child, wilfully or accidentally jump or fall out of a window, physical injury is the consequence; and in this case, as a general rule, the certainty of the penalty suffices to make men conform to the laws of nature. But the penalties inflicted by society are voluntary on its part, and awarded under a sense of responsibility; for, as Baron Bramwell remarks, pain being in itself an evil, we are only justified in occasioning it to another for some good and substantial reason; in other words, we punish, because the balance of convenience is in favour of punishment for the repression of crime. The law itself fully recognises this principle, in the gradations of punishment which it prescribes for the same offence, and the application of which it leaves to the Judge. There is no mere blind penalty attaching to the infraction of any law; the circumstances of each case are to be taken into account—the amount of temptation, the position and opportunities of the criminal, and the consequences of his crime. To make murder an exception to these principles is impossible, because the punishment of murder is inflicted under just the same kind of responsibility as the most trifling imprisonment. It is not the mere act of depriving another of life that in practice ensures the penalty of death—for even those who consider that the putting of a murderer to death is based upon a direct divine command, would no more execute an idiot than Baron Bramwell himself. You have not, therefore, and cannot have, a *certainty* that even the highest crime known to the law shall, on the conviction of the perpetrator of it, be followed by the exaction of the highest penalty. To hang an acknowledged idiot would, under any circumstances, so shock the public mind, that it would be considered as tantamount to deliberate murder. Here, then, lies the very gist of the subject. It being shown, then, that the penalty even of murder does not follow on its commission as a matter of logical and inevitable necessity, like the penalties attaching to violations of physical laws, the question arises, Who are idiots? and why are they to form the subjects of this exception?

This is an inquiry more easily made than satisfactorily answered; and yet it must be answered, if jurisprudence is to have the slightest claim to be regarded as a science.

Idiots, as is well known, vary in their salient characteristics. Some of them, besides being destitute of the smallest glimmerings of reason, are also sunk into the lowest depths of physical disability. "Sans teeth, sans eyes, sans taste, sans everything," they are apparently wholly without mind—they could not, therefore, commit murder, from their state of bodily infirmity, even if mentally they could conceive the crime. Other idiots, again, like to the former class as to their mental powers, are yet endowed with great bodily strength, so that they may become exceedingly dangerous to those whom they dislike, and are known not unfrequently to have deliberately murdered them. For what reason, then, are they exempted from execution? Plainly because of the condition of their minds. Where, then, is the line to be drawn which separates the imbecile who forms a fit subject for execution, from the imbecile whose execution would be considered as a murder?

Notwithstanding the dicta of the Judges in the House of Lords, in the case of MacNaughten, it will be found that practically there are no general rules on the subject—each case is in reality determined on its real or supposed merits. Juries may be misled into giving a verdict—guided they are not—by what is told to them about a knowledge of right and wrong on the part of the prisoner; but they convict or acquit him just according to their own preconceived notions of insanity. If they believe the accused person to be so mad that he would have formed a fit inmate of a lunatic asylum, they acquit him on the ground of insanity, without ever troubling their heads as to whether he knew the distinction between right and wrong, or was or was not conscious that he was doing a criminal act when he committed the murder. In this they act rightly, and in accordance with the dictates of common sense, guided by their ideas of moral responsibility to the Almighty—for, as every one knows, except perhaps lawyers, the test of a knowledge of right or wrong is utterly fallacious. The maddest lunatic confined in Bedlam acts from motives, and does wrong knowing that it is wrong, just like any sane and reasoning sinner.

In cases in which the test that juries apply in their own minds has already been brought to a practical issue, and the accused person is confined in an asylum, a conviction for murder is impossible, no matter how deliberate and cruel the circumstances attending it may have been. Amongst the blackest murders that have ever been committed, are those perpetrated by the inmates of asylums; yet they go unpunished, because the moral sense of mankind revolts against the punishment of a being deprived of the guidance of reason. Who will deny that there are beings going freely about the world who are just as mad as others who are under restraint in asylums? Why, then, is a clumsy test of this kind, which depends upon the local and peculiar circumstances of the lunatic, to form a guide in the case of an issue so awful as that of life and death? Because a poor creature is so unfortunate as to have no friends able or willing to take the somewhat complicated steps necessary for securing him in an asylum, is that any reason why, when he commits a crime, he is to be subjected to penalties which humanity forbids to be applied to his brother lunatics in asylums? We put the question in this way, not because we do educated men the injustice of supposing that they would knowingly sanction any such doctrine as that referred to, but in order to show that there is an inherent necessity that each case, where insanity is pleaded as an excuse, shall be judged and disposed of exclusively upon its own individual merits; and we have the further object of showing that it is because the test supplied by the law—the knowledge of right and wrong—is insufficient, that juries take these matters into their own hands, and often acquit prisoners in the teeth of the directions of the presiding Judge.

Having got thus far, we are now in a position to answer the question as to what should be the course to be practically adopted in these distressing cases; and we have no hesitation in expressing an opinion that the same species of test should be applied here as is by law imperative before any man can be consigned to the custody and refuge of an asylum—viz., an investigation into the mind of the alleged lunatic by scientific examiners. The facts of the case are matters for the jury to determine; the law and the facts are for the Judge; but the question of the infliction of the appropriate penalty should be for the executive, aided by skilled witnesses, or, as they are called on the Continent, "Peritii."

A rule of this kind works admirably in the State of Maine. In cases of insanity alleged as an excuse for criminal acts, the person implicated is remanded to safe custody, and carefully watched and examined by eminent medical men, who have made the diseases of the mind subjects of special study, and are appointed to their office by the State. At the expiration of a certain time, having taken into their consideration the facts proved in evidence, and associated them with the results of their own observations, they make their report, and upon that report the defence of insanity stands or falls. If a similar plan were adopted in this country, there can be little doubt in the minds of those who take an enlarged view of the subject, that the administration of justice would be rendered much more certain than it is at present; defences grounded on presumed insanity would be less frequent, because they could never prevail except upon just and equitable grounds; the penalties of the law would be inflicted upon intelligible data, and, by commanding the assent of the public mind, would act to a far greater extent as preventive spectacles than they do now.

What can be more disgraceful to a civilized community than that the most subtle of all questions, the sanity of a human being, should be left to the decision of a petit jury, composed of men who are often as illiterate as they are prejudiced, and who are guided much more by feeling than by reason? And this leads us to remark, in conclusion, that great injustice is often done to prisoners by the way in which these subjects are argued at the bar. Instead of trusting to the truth, and carefully making themselves masters of the facts of the case, counsel who undertake to prove the insanity of a prisoner are too often in the habit of treating the whole matter as one of sentiment, which it is not at all, and of making compassionate appeals to the feelings of the jury, in the hope of averting the capital sentence. It can hardly be wondered at that, under these circumstances, the feelings of the bench and the public are excited against defences on the ground of insanity, and that they come to regard them merely as tricks of counsel, analogous to the technical pleas of a special pleader. We trust, however, that, through the medium of the Juridical Society, the Judges and the Bar may now be led to regard the question in its philosophical bearings; and we entertain no fears of the result, so soon as they begin to argue it as a logical problem, to be determined by considerations of abstract justice, based upon experience, and guided by a sense of moral responsibility.

